# Diagnostic Results of IQ-test in School-Aged Children with Fetal Alcohol Syndrome and Fetal Alcohol Spectrum of Disorders

**DOI:** 10.1192/j.eurpsy.2022.1068

**Published:** 2022-09-01

**Authors:** E. Fadeeva, A. Nenasteva

**Affiliations:** Federal State Autonomous Educational Institution of Higher Education I.M. Sechenov First Moscow State Medical University of the Ministry of Health of the Russian Federation (Sechenov University), Institute Of Clinical Medicine N.a. N.v. Sklifosovsky, Moscow, Russian Federation

**Keywords:** School-Aged Children, Fetal Alcohol Syndrome, Fetal Alcohol Spectrum of Disorders

## Abstract

**Introduction:**

FAS and FASD are completely preventable conditions which can be reduced by methods of prevention aimed at alcohol eliminating by women during pregnancy.

**Objectives:**

The aim of the study was to assess level of intellectual impairment in children with FAS and FASD.

**Methods:**

All children who participated in the study had physical development retardation and various dysmorphological features of FAS or FASD. The sample included 77 children, 8.6±1.03 years of age. FAS was diagnosed in 8 children, FASD in 69 children. Assessments were carried out by pediatrician, psychiatrist and psychologist; level of intelligence was assessed using WISC test.

**Results:**

Among children with FAS average IQ was 65.9 points (extremely low level), which corresponds to «mild mental retardation» diagnosis (F70, ICD-10). Four children with FAS had intelligence corresponding to «very low» level (IQ=70-79), three had «mild mental retardation» (IQ=50-69), and one had «moderate mental retardation» (F71, ICD-10) (IQ=35-49). Among children with FASD average IQ was higher and reached 79.5 points, corresponding to «very low» intelligence level. «Moderate mental retardation» was identified in 7.8% children with FASD; 22.1% children had «mild mental retardation», and 27.3% had «very low». In 37.7% children IQ level was within normal range: «low average» in 19.5% (IQ=80-89) and «average» in 18.2% (IQ=90-109). «Very high intelligence» (IQ=120-129) was detected in 2.6% children, «extremely high intelligence» (IQ=130 points and above) in 2.6%.

**Conclusions:**

All children with FAS had impaired mental development. Children with FASD showed a wide range of total IQ values, from moderate degree of mental retardation to very high intelligence.

**Disclosure:**

No significant relationships.

